# The application of breath-holding in sports: physiological effects, challenges, and future directions

**DOI:** 10.1007/s00421-025-05752-y

**Published:** 2025-03-24

**Authors:** Antonis Elia, Frédéric Lemaître

**Affiliations:** 1 Division of Environmental Physiology, Department of Physiology and Pharmacology, Karolinska Institute, Berzelius väg 13, Solna, 171 65 Stockholm, Sweden; 2 DevAH UR n°3450, Faculty of Sports Sciences, University of Lorraine, Nancy, France

**Keywords:** Breath-hold, Erythropoietin, Exercise performance, Haemoglobin, Hypoxic training, Spleen

## Abstract

Repeated breath-holding has been shown to elicit transient increases in haemoglobin and erythropoietin concentrations, while long-term engagement in breath-hold-related activities has been linked with improved hypercapnic tolerance, mental resilience, and favourable cardiorespiratory, cerebrovascular, and skeletal muscle adaptations. Given these findings, breath-holding was proffered as a possible performance optimisation strategy a little over a decade ago. This prompted practitioners and researchers to explore its broader application either as a priming strategy completed immediately before an endurance activity or as an alternative hypoxic-hypercapnic training method. Therefore, this review aims to offer an update of the acute and long-term physiological responses to breath-holding that are relevant to athletic performance and provide an overview of the existing body of knowledge surrounding its potential utility and efficacy as a performance enhancement strategy. Current evidence suggests that breath-holding may have potential as a priming strategy; however, further placebo-controlled studies are required to rigorously evaluate its efficacy. Additionally, it is evident that developing an effective protocol and administering it successfully is more complex than initially thought. Key factors such as the characteristics of the prescribed protocol, the timing of the intervention relative to the event, and the nature of the existing warm-up routine all require careful consideration. This highlights the need for adaptable, context-specific approaches when integrating breath-holding into real-world sporting environments. Finally, while dynamic breath-hold training shows the greatest potency as a performance optimisation strategy, further research is necessary to determine the optimal training protocol (i.e., hypoxaemic-hypercapnic dose), and duration.

## Introduction

Breath-hold physiology has captivated the scientific community’s interest for over a century (Hill and Flack 1908), with a substantial body of literature outlining the morphological characteristics of competitive and habitual diving populations as well as the physiological responses occurring during and/or shortly after prolonged breath-holding (e.g., the diving response, trigeminocardiac reflex, haematological responses, etc.) (see reviews by Lin 1988; Gooden 1994; Ferretti 2001; Ferretti and Costa 2003; Foster and Sheel 2005; Fitz-Clarke 2018; Elia et al. 2021c). This extensive corpus of research has shed light into the intricate mechanisms underlying breath-holding and provided a glimpse into the long-term adaptive physiological processes associated with it.

Breath-holding performed in a sequential manner has been shown to elicit transient increases in haemoglobin (Schagatay et al. 2001; Elia et al. 2021b) and erythropoietin concentrations (de Bruijn et al. 2008; Elia et al. 2019a, 2021a), while long-term engagement in breath-hold-related activities has been linked with a blunted ventilatory response to hypercapnia (Delapille et al. 2001; Grassi et al. 1994; Roecker et al. 2014; Song et al. 1963), mental resilience (Alkan and Akis 2013; Allinger et al. 2024b), and favourable cardiorespiratory (Costalat et al. 2017, 2015; Lemaitre et al. 2015), cerebrovascular (Joulia et al. 2009; Moir et al. 2019; Vestergaard and Larsson 2019), and skeletal muscle adaptations with respect to performance (Bae et al. 2003; Kjeld et al. 2018; Elia et al. 2019b). Taken together, the insights gleaned from the literature led practitioners and researchers to explore the broader application of breath-holding as either a priming strategy prior to endurance events or as an alternative hypoxic-hypercapnic training modality (see reviews by Lemaitre et al. 2010; Bouten et al. 2024).

In this review, we aim to provide an update relating to the physiological effects associated with acute and long-term engagement in breath-hold-related activities relevant to athletic performance, delve into breath-hold priming strategies and training regimens used to improve performance, but also explore how these as well as breath-holding per se could effectively and safely be applied across different sports and athletic pursuits. Duly, this review is intended to help inform practitioners, coaches, athletes and researchers about the possible effects, challenges and potential applications of breath-hold training and the necessary precautions that ought to be in place when employed.

## Physiological responses to acute breath-holding

In humans, the theoretical maximum breath-hold duration following air breathing (21% oxygen) is determined by the body’s oxygen reserves and the rate at which they are consumed (Ferretti et al. 1991; Mithoefer 1959, 1965). Since aerobic metabolism during a breath-hold is limited to the body’s finite oxygen stores, a larger initial oxygen reservoir will extend the aerobic dive limit, thereby enabling longer breath-holds to be reached (Mithoefer 1959; Whitelaw et al. 1987). Thus, factors that may contribute towards enhancing the body’s oxygen reservoirs are considered advantageous with respect to breath-hold performance.

Unlike diving mammals which possess exceptionally high oxygen stores in their skeletal muscle and blood—both of which are key predictors of their diving capabilities—human breath-hold capacities are greatly dependent on lung oxygen stores. These reserves are influenced by the inspired alveolar oxygen fraction and lung volume, as for any given oxygen fraction in the alveoli, an individual’s lung volume is directly proportional to their oxygen stores (Mithoefer 1965; Muxworthy 1951; Whitelaw et al. 1987). It is thus, not surprising that breath-hold performance significantly improves when conducted at higher lung volumes [e.g., in proximity to total lung volume (TLC) vs. at ~ 85% of lung volume] (Overgaard et al. 2006; Whitelaw et al. 1987; Mithoefer 1965). These performance gains result from a greater oxygen reservoir being readily available to support aerobic metabolism but also from an attenuated oxygen desaturation rate, an enhanced carbon dioxide buffering capacity, and a delayed onset of the Hering–Breuer deflation reflex (Godfrey et al. 1969; Mithoefer 1959, 1965; Rose et al. 1979). Altogether, underscoring the elemental role of oxygen stores in determining breath-hold capacity.

### Cardiovascular responses

**Fig. 1:**
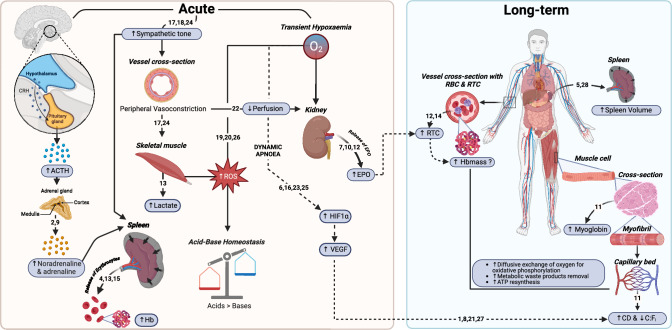
A schematic overview of current knowledge on the acute and long-term physiological responses to breath-holding. Arrows up (↑) and down (↓) within framed box indicate an increase or a decrease of the associated variable. Dotted arrow lines (-----) indicates potential mechanisms. ACTH, adrenocorticotropic hormone; ATP, adenosine triphosphate; C:F_i_, capillary-to-fibre ratio; CD, capillary density; CRH, corticotropin-releasing hormone; EPO, erythropoietin; Hb, haemoglobin concentration; Hbmass, haemoglobin mass; HIF1a, hypoxia-inducible factor 1 alpha; O_2_, oxygen; RBC, red blood cells; ROS, reactive oxygen species; RTC, reticulocyte count. Supporting literature is denoted by numbers where; 1 = Arany et al. (2008), 2 = Ayers et al. (1972), 3 = Baković et al. (2003), 4 = Bakovic et al. (2013), 5 = Bouten et al. (2019), 6 = Breen et al. (1996), 7 = de Bruijn et al. (2008), 8 = Desplanches et al. (1993), 9 = Eichhorn et al. (2017), 10 = Elia et al. (2019a), 11 = Elia et al. (2019b), 12 = Elia et al. (2021a), 13 = Elia et al. (2021b), 14 = Engan et al. (2013), 15 = Espersen et al. (2002), 16 = Gustafsson and Sundberg (2000), 17 = Heistad et al. (1968), 18 = Heusser et al. (2009), 19 = Joulia et al. (2002), 20 = Joulia et al. (2003), 21 = Kon et al. (2014), 22 = Kyhl et al. (2016), 23 = Rodriguez-Miguelez et al. (2015), 24 = Sterba and Lundgren (1988), 25 = Stroka et al. (2001), 26 = Sureda et al. (2015), 27 = Terrados et al. (1990), 28 = Yang et al. (2022). Figure created with BioRender.com

During the state of breath-holding, a series of physiological responses are elicited. Amongst these, a parasympathetically-induced bradycardia is noted, caused by the removal of the phasic tachycardia during inspiration and the pulmonary stretch receptor input converging at the *nucleus tractus solitarius* (Kato et al. 1988; Lin et al. 1974; Hayashi et al. 1997; Lemaitre et al. 2015); the teleological benefit of which is to lower the myocardial oxygen consumption (Hoiland et al. 2017). Peripheral vasoconstriction is initiated via an elevated sympathetic tone at the body’s extremities and non-vital organs (Sterba and Lundgren 1988; Heusser et al. 2009; Breskovic et al. 2011; Heistad et al. 1968; Leuenberger et al. 2001), with these shifting from primarily aerobic to predominantly anaerobic metabolism (Fig. 1). The functional role of the peripheral vasoconstriction is to prioritise oxygen-rich blood to the brain as attested by the ensuing rise in carotid artery (Jiang et al. 1994; Pan et al. 1997) and cerebral blood flow (Joulia et al. 2009; Vestergaard and Larsson 2019). Overall, the primary function of these physiological responses is to slow the rate of oxygen desaturation until respiration is restored.

Though evident in all humans, the magnitude of the physiological components discussed above vary greatly between diving and non-diving cohorts, differences which may result from training-induced stimuli and/or a natural selection of genetic polymorphisms (see review by Elia et al. 2021c). Regardless, it is evident that when breath-holding is coupled with face immersion, the bradycardial response is potentiated in both populations, resulting in longer breath-holds to be attained (Schuitema and Holm 1988; Hayashi et al. 1997; Daly 1997; Perini et al. 2010). Thus, when the forehead, eyes, and nose come into contact with water, the facial cold receptors—which are innervated by the ophthalmic nerve—are activated (Schagatay and Holm 1996). This interaction triggers the trigeminal nerve, which converges with the motor nucleus of the vagus nerve, consequently evoking the ‘trigeminocardiac reflex’ (Hayashi et al. 1997; Lemaitre et al. 2015). This reflex is characterised by bradycardia, hypotension, gastric hypermobility and cerebrovascular vasodilatation (Schaller 2004; Lemaitre et al. 2015). Importantly the extent of this reflex is highly variable and primarily depends on the water temperature the facial cold receptors are exposed to, with water temperatures between 10 and 15 °C eliciting a more pronounced response (Daly 1997), while temperature variations above this range seem to have minimal effect (Schagatay and Holm 1996; Asmussen and Kristiansson 1968; Mukhtar and Patrick 1986; Folinsbee 1974). Thus, the combination of breath-holding and face immersion has a synergistic effect on bradycardia, which is greater than the sum of each individual response (Marsh et al. 1995; Hayashi et al. 1997).

### Haematological responses

#### Splenic contractions

The spleen represents a constitutive part of the sympathetic nervous system (Felten et al. 1985), is implicated in the process of erythrophagocytosis, and serves as an antibody production site as well as an erythrocyte reservoir, with humans roughly storing 10% of their total erythrocyte volume within it (Stewart and McKenzie 2002). Under conditions where the sympathetic nervous system is stimulated (e.g., exercise, hypercapnia, hypoxaemia), the spleen contracts, concurrently releasing its stored erythrocytes into the systemic circulation (Laub et al. 1993; Shephard 2016; Elia et al. 2021b, 2024b; Pernett et al. 2021; Lindblom et al. 2024) (Fig. 1). In a breath-holding context, the splenic response has been documented to reach full effect after a series of 3–5 repeated maximal breath-holding attempts, with haemoglobin increases of 3–8 g/L being measured in untrained cohorts (Schagatay et al. 2001, 2005; Richardson et al. 2005; Elia et al. 2021b, 2022a). These increases potentiate the oxygen binding and carrying capacity of blood; hence, the oxygen reserve is increased by the systemic mobilisation of erythrocytes. Notably, these elevations are reported to be restored to pre-breath-holding levels within a 10-min window (Schagatay et al. 2001, 2005). As a result, successive breath-holds begin with a greater amount of readily available oxygen, attenuating the oxygen desaturation rate, and thereby contributing to an extended breath-hold duration (Bakovic et al. 2013; Schagatay et al. 2005). Thence, a larger splenic volume, capable of storing a greater number of erythrocytes is considered advantageous for breath-holding performance (Elia et al. 2021b; Schagatay et al. 2012).

### Erythropoietin

Exposure to hypoxic/hypoxaemic conditions activates the hypoxia-inducible factor (HIF) signalling, consequently promoting the transcriptional activation of the erythropoietin (EPO) gene (Ebert and Bunn 1999; Haase 2013, 2010). This, in turn, stimulates the production of the glycoprotein hormone EPO, primarily by the kidneys (Jelkmann 1992; Knaupp et al. 1992). While no studies have yet explored the effects of voluntary breath-holding on HIF expression, several investigations have demonstrated that the systemic hypoxaemia brought on by breath-holding transiently elevates serum EPO levels (de Bruijn et al. 2008; Elia et al. 2019a, 2021a; Kjeld et al. 2015). Specifically, in both trained (Elia et al. 2019a) and non-divers (Elia et al. 2021a; de Bruijn et al. 2008), a series of repeated breath-holds (5–15 repetitions) has been shown to be efficacious in stimulating the release of the glycoprotein hormone EPO, with these increases being notably greater after a series of dynamic [+ 4.0 mIU/L (+ 63%), Elia et al. (2019a)] as opposed to static breath-holds [+ 1.4 mlU/L (+ 16%), de Bruijn et al. (2008)]. Considering that the magnitude of EPO release is directly proportional to the level of systemic hypoxemia (Elia et al. 2019a; Eckardt et al. 1989; Knaupp et al. 1992), it is perhaps unsurprising that dynamic breath-holds led to a higher EPO increase, since this protocol was associated with a more pronounced desaturation [62 ± 10% (Elia et al. 2019a) *vs*. 73 ± 11% (de Bruijn et al. 2008)].

### Breath-hold phases

Inevitably, over the time course of a voluntary breath-hold, the arterial partial pressure of oxygen gradually decreases while carbon dioxide levels rise (Lin et al. 1974). These changes stimulate both central and peripheral chemoreceptors, consequently increasing the ventilatory drive and respiratory distress experienced by the apnoeist (Feiner et al. 1995). Based on these sensory chemoreflexes, a breath-hold can be divided into two distinct phases, namely: the “easy-going” and the “struggle” phase, separated by the physiological breaking point, identified as the point where the first involuntary breathing movement (IBM) is noted (Agostoni 1963; Lin et al. 1974). During the easy-going phase, the subject feels no immediate urge to breathe. In contrast, the struggle phase is defined by the individual’s psychological tolerance to the increasing hypoxaemic and hypercapnic stress, as well as the progressively intensified IBMs (Lin et al. 1974). Therefore, although the former can be quantified by physiological means (e.g., hypoxaemia and hypercapnia) (Feiner et al. 1995), the latter is as equally determined by volitional factors (Schneider 1930; Rigg et al. 1974; Lin et al. 1974).

In summary, the ability to suppress respiratory urges and sustain breath-holds for prolonged durations relies on three fundamental principles: (*i*) the capacity for oxygen storage [(i.e., lungs, blood (haemoglobin) and skeletal muscle (myoglobin)], (*ii*) the efficacy of oxygen conservation and utilisation (i.e., in large part dependent on cardiovascular and metabolic adjustments), and (*iii*) volitional factors (i.e., psychological tolerance to the increasing urge to breathe and the continuously intensified IBM).

## Athletic performance and breath-holding

Under normoxic conditions, the maximal rate at which oxygen can be transported from the environment to the mitochondria and utilised to support oxidative phosphorylation is widely recognised to be determined by the physiological limits of the Fick equation (Levine 2008). Maximal oxygen uptake ($\dot{\text{V}}$O_2max_), one of the major characteristics that determine performance in endurance sport (di Prampero 2003), is attained through a concurrent increase in cardiac output ($\dot{\text{Q}}$) and the arteriovenous oxygen content difference (Wasserman 2005). In endurance-trained athletes, oxygen transport is the main limiting factor of $\dot{\text{V}}$O_2max_, with an estimated 70–80% attributed to maximal $\dot{\text{Q}}$, whereas in untrained individuals, mitochondrial oxygen consumption also plays a major role (Levine 2008; Wagner 2000; Cerretelli and Di Prampero 1987). Therefore, increasing the oxygen supply to the exercising muscles might thus improve performance (Mallette et al. 2018; Bejder et al. 2019; Linnarsson et al. 1974; Macdonald et al. 1997).

The oxygen carrying capacity of blood is primarily facilitated by haemoglobin, the main protein in erythrocytes. Haemoglobin transports ~ 98% of the oxygen in blood, with the remaining ~ 2% (0.3 mL oxygen per 100 mL of plasma) dissolved and carried in plasma (Pittman 2011; Dunn et al. 2016). When fully saturated and assuming a normal haemoglobin concentration (e.g., 140 g L^−1^) with a constant oxygen capacity (1.39 mL g^−1^), haemoglobin carries nearly 20 mL of oxygen per 100 mL of whole blood (McArdle et al. 2010). Given this, it is not surprising that variations in blood volume, total haemoglobin mass (t-Hb_mass_) and haemoglobin concentration lead to reciprocal changes in exercise capacity, proportional to alterations in the oxygen-carrying capacity of blood (Kanstrup and Ekblom 1984; Parisotto et al. 2000; Prommer et al. 2007; Calbet et al. 2006; Bejder et al. 2019). Among these parameters, t-Hb_mass_ (r = 0.79) exhibits the strongest relationship with $\dot{\text{V}}$O_2max_ (Schmidt and Prommer 2008; Convertino 1991; Sawka et al. 2000; Kanstrup and Ekblom 1984). Specifically, a 1% change in t-Hb_mass_ was associated with a 0.6–0.7% change in $\dot{\text{V}}$O_2max_ (Saunders et al. 2013). Consequently, factors that may contribute towards enhancing the availability of oxygen, such as the level of circulating haemoglobin, are considered advantageous from a performance perspective (Heinicke et al. 2001; Schmidt et al. 2002; Calbet et al. 2006).

### Breath-holding as a priming strategy

In the ever-ending search for performance optimisation strategies, a little over a decade ago, breath-holding was proffered as a potential candidate (Lemaitre et al. 2010), as of its capacity to transiently elevate haemoglobin concentrations and t-Hb_mass_ through a splenic response (Keeler et al. 2024; Schagatay et al. 2001; Elia et al. 2021b). More importantly, these increases are reported to persist up to 10-min after breath-holding (Schagatay et al. 2001, 2005). As such, the ability to acutely improve, by natural means, the oxygen binding and carrying capacity of blood allured researchers to explore the application of breath-holding as a priming strategy prior to endurance events.

Sperlich et al. (2015) was amongst the first to put this hypothesis to test. In their study, the authors found that when a cycling time-trial was preceded by breath-holding (i.e., four repeated dry maximal bouts), as opposed to eupnoea, it took on average 16 s longer for the subjects to reach the 4-km endpoint [(breath-holding) 342 ± 34 s *vs.* 326 ± 68 s (control)] (Table 1). At face value, these results suggested that breath-holding may not serve as an effective priming technique. However, a couple of methodological limitations merit consideration. For instance, their subjects terminated their breath-holds before any form of desaturation occurred [peripheral oxygen saturation (SpO_2_), (pre) 98 ± 0% *vs.* 99 ± 0% (post)], possibly attributed to the lack of familiarisation to breath-holding, consequently the hypoxaemic stimulus was inadequate to trigger the splenic response [(pre) 71 ± 10 mL *vs.* 63 ± 11 mL (post)]. Additionally, the time-interval between the last breath-hold and the time-trial may have been too short (30–45 s) to ensure complete recovery from the mental challenges posed by serial breath-holding—a notion supported by the split times of the 4-km time-trial.

**Table 1:**
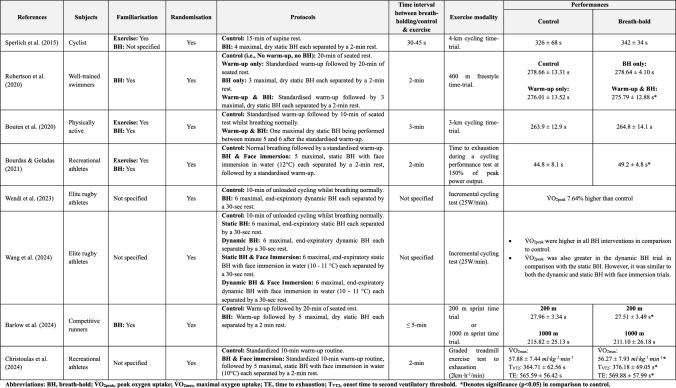
Available studies that examined the use of breath-holding as a priming strategy for exercise performance

To rectify these limitations, subsequent work by Robertson et al. (2020) and Bouten et al. (2020) incorporated a familiarisation session, and extended the time-interval between the breath-holds and the exercise trial (2–3 min). Interestingly, Robertson et al. (2020) noted an earlier time-to-completion when the 400-m freestyle time-trial was performed after a standardised warm-up and serial breath-holding (275.79 ± 12.88 s) compared with breath-holding-only (278.64 ± 4.10 s) and control (278.66 ± 13.31 s; i.e., no warm-up nor breath-holding), results that did not stem from haemoglobin level differences. Yet, similarly to the study of Bouten et al. (2020), no differences were discerned with the warm-up-only trial (Table 1), conjointly reiterating the elemental role of warm-up. Now given that exercise independently evokes splenic contractions (Laub et al. 1993; Stewart et al. 2003; Lindblom et al. 2024), using breath-holding for the sole purpose of stimulating the splenic response seems unnecessary, especially when a warm-up is feasible.

More recently, Bourdas and Geladas (2021) demonstrated that a series of five repeated maximal static breath-holds combined with face immersion in cold water (12 °C) followed by a 150 s warm-up (i.e., 1 W per kg of body mass load at 50–75 rpm ad libitum) successfully improved time to exhaustion (49.2 ± 4.8 s *vs.* 44.8 ± 8.1 s) during a cycling test [(i.e., intensity corresponding to 150% of peak power output (PPO)]. The reported performance gains likely pertained to a higher (~ 50%) accumulated V̇O_2_ over the time course of the exhaustion test; a response that, similarly to Robertson et al. (2020), occurred in absence of any breath-hold-related changes in haemoglobin levels (Table 1). The authors tentatively ascribed their results to a disturbance of the acid–base homeostasis, offering, for the first time, a potential alternative way by which serial breath-holding may act to affect performance. Later work by Wendi et al. (2023) indicated that integrating six maximal breath-holds over a 10-min unloaded cycling warm-up routine significantly improved peak oxygen uptake ($\dot{\text{V}}$O_2peak_) during an incremental cycling test (Table 1). Even though they did not evaluate acid–base responses, they too did not record any changes in haemoglobin nor in red blood cell levels after breath-holding. Overall, it appears that beyond the splenic response, there seem to be other, possibly more potent mechanisms through which breath-holding may affect exercise performance, underscoring the need for further research.

The application of dynamic breath-holding combined with face immersion, has also recently been explored (Wang et al. 2024). Integrating six maximal end-expiratory breath-holds, each separated by 30 s of normal breathing, over a 10-min unloaded cycling warm-up routine significantly improved V̇O_2peak_ during a subsequent incremental cycling test (Wang et al. 2024). Importantly, these gains were greater than those registered after eupnoea (i.e., normal breathing and unloaded cycling) and dry static breath-holds (i.e., no unloaded cycling), yet these were comparable to the improvements seen when dynamic and static breath-holds were combined with face immersion (Table 1).

In summary, current evidence does not definitively support or refute the effectiveness of breath-holding as a priming strategy, highlighting the need for additional research. Of notable interest, however, is that most studies reporting favourable outcomes employed experimental designs with greater number of breath-holds (≥ 5 repetitions), coupled with physical exercise and/or face immersion (Table 1); combinations known to augment hypoxaemic and hypercapnic stress. Ergo, future research should focus on exploring these combinations further and pursue to elucidate the underlying mechanisms through which these may act to influence exercise performance. More importantly, given the impact of placebo and nocebo on exercise performance (Hurst et al. 2020; Beedie et al. 2018), it is essential for future research to incorporate placebo-controlled trial(s) into their experimental designs. This approach would allow to more adequately evaluate the efficacy of breath-holding as a priming strategy, clarifying whether performance improvements arise from the intervention itself or from psychobiological responses to perceived beneficial interventions (placebo) or adverse expectations (nocebo).

### Practical applications and challenges

The potential utility of breath-holding as a priming strategy for acute performance optimisation is propitious, yet constructing a potent protocol, and administering it effectively is proving more complex than initially thought, with several aspects necessitating careful rumination. Accordingly, the next section will outline factors that should be taken into consideration when devising breath-holding protocols for athletic activities.

#### Structure

**Fig. 2:**
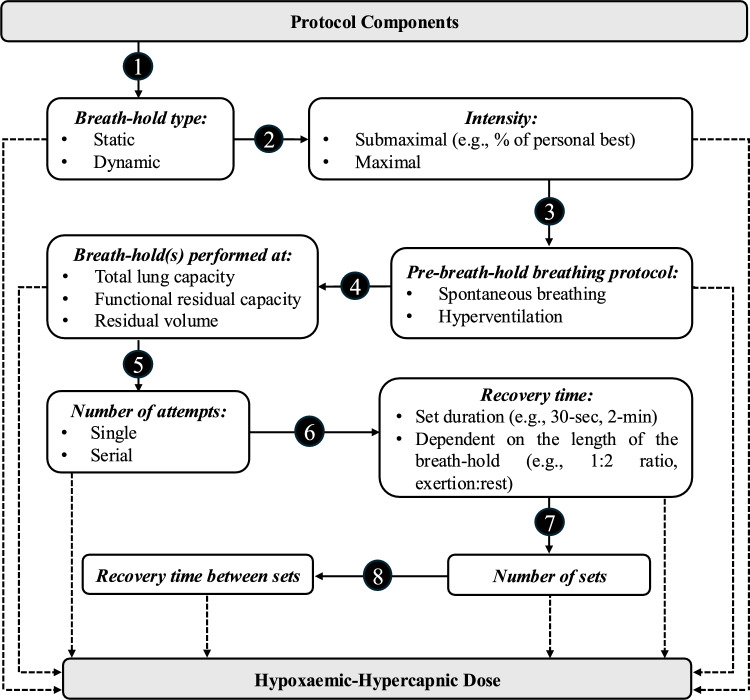
Schematic representation illustrating the eight distinct layers that constitute a breath-holding protocol

A breath-holding protocol comprises of eight distinct parts, each requiring careful consideration, as they will collectively determine the volume and duration of the intervention (Fig. 2). These consists of the: (*i*) type of breath-holding (i.e., static or dynamic), (*ii*) intensity (i.e., maximal or submaximal), (*iii*) pre-breath-hold breathing protocol (i.e., spontaneous breathing, hyperventilation), (*iv*) lung volume at which the attempt is performed (i.e., functional residual capacity, total lung capacity, residual volume), (*v*) number of attempts (i.e., single or serial), (*vi*) recovery time between each attempt (e.g., ≤ 120 s), (*vii*) number of sets, and (*viii*) resting period between each set. Decisions across all levels should be informed by the athletes’ physical capabilities and adjusted based on their responses, as if the protocol is, for instance, too intense it may lead to residual fatigue, subtly undermining performance. It should be noted, however, that the effects of different breath-holding practices/combinations on performance are unknown, emphasising the need for additional research to guide optimal protocol design.

#### Mental fatigue

A seldom-discussed and frequently overlooked component within the literature is the impact of breath-holding on mental and psychological strain. Although current evidence may advocate for physiologically challenging protocols to be implemented, psychological aspects should also be considered (e.g., physical/mental exertion and performance readiness). To exemplify, Bouten et al. (2020) showed that a solitary static breath-hold was rated more favourably in terms of perceived exertion and performance readiness than a single dynamic breath-hold or a series of repeated breath-holds (i.e., 5–6 bouts). Despite being auspicious, when introduced after a 10-min warm-up, it failed to improve subsequent performance. Likewise, compared with control, when looking at the split times of the 4-km time-trial in Sperlich et al. (2015) study, cyclists lost 7 s during their first kilometre, indicating that repeated breath-holding may have induced a degree of fatigue. It is, therefore, evident that when devising breath-holding protocols, one must carefully weigh physiological and psychological factors, as prescribing overly strenuous protocols could adversely affect endurance performance, while excessively weak ones may offer no discernible effects.

#### Warm-up routines and breath-holding

Warming-up prior to a competitive exercise bout is a widely accepted practice in the modern sporting environment; acknowledged as essential for optimising performance (McGowan et al. 2015). Both passive and active warm-up routines can evoke temperature, metabolic, neural and psychology-related effects, including increased anaerobic metabolism, improved oxygen uptake kinetics and post-activation potentiation (Sale 2002; Gray and Nimmo 2001; Pearce et al. 2012; Poole and Jones 2012). Accordingly, breath-holding should not be viewed as a replacement to traditional warm-up routines; rather, it should be explored as a prospective adjunct to these. In support of this, emerging evidence indicate positive performance outcomes when breath-holding was incorporated either before (Bourdas and Geladas 2021; Christoulas et al. 2024), after (Robertson et al. 2020; Barlow et al. 2024), or even during (Wendi et al. 2023; Wang et al. 2024) their respective warm-up regimens. These findings signify the potential use of breath-holding as a supplementary tool in optimising athletic performance. From a practical standpoint, however, dry dynamic breath-holds, may be more feasible for athletes to incorporate into their warm-up routines due to their simplicity and minimal equipment needs, making them easier to adapt in a variety of exercise settings.

#### Other situational factors

While breath-holding is seemingly easy to introduce prior to an endurance activity, since it does not require access to any specialised equipment, the question remains whether it can seamlessly be integrated into real-world sporting environments. In this regard, the nature of the existing warm-up routine (e.g., format, duration, intensity), type of event that follows (i.e., training or competition) as well as any constrains imposed by the competition (i.e., time available for warm-up, time-interval between warm-up and competition) are additional elements that need to be factored in when devising and prescribing breath-holding protocols. For example, the timing of the breath-holding intervention relative to the event is crucial, as a significant time delay between these may negate any potential effects. Altogether, emphasising the need for adaptable and context-specific approaches to integrating breath-holding into athletic preparation.

## Long-term effects of breath-hold training

The hypoxaemic hypercapnic nature of breath-holding, along with its associated physiological responses—such as the transient increases in EPO—as well as the long-term physiological and psychological adaptations derived from chronic engagement in breath-hold-related activities (Elia et al. 2021c), have led researchers to investigate whether breath-hold training could serve as a viable alternative training modality for performance optimisation (Lemaitre et al. 2010). In accordance, the following section will offer an overview of the long-term effects of breath-hold training, its potential application(s) and, where possible, its impact on athletic performance.

### Haematology

Breath-holding performed in a serial manner and interspersed with short periods of normoxic breathing (i.e., 2-min) has been shown to transiently elevate EPO concentrations (see Sect. 2.2.2.). Given the pivotal role of EPO in the process of erythropoiesis (Jelkmann 1992; Elliott 2008), and the strong relationship between t-Hb_mass_ and V̇O_2max_ (Sawka et al. 2000; Saunders et al. 2013), several studies have sought to elucidate the long-term effects of breath-hold training on haematology (Table 2).

**Table 2:**
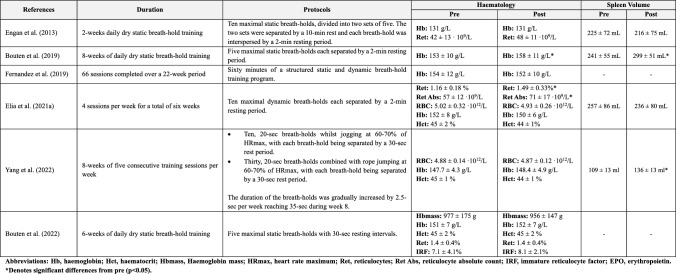
The effect of breath-hold training on haematology and spleen volume

While some studies have documented responses suggestive of active erythropoiesis (i.e., increases in reticulocytes, iron levels and reductions in ferritin) (Engan et al. 2013; Elia et al. 2021a), none have yet revealed discernible differences in red blood cell count at rest (Elia et al. 2021a; Yang et al. 2022; Astolfi et al. 2022), nor t-Hb_mass_ (Astolfi et al. 2022; Bouten et al. 2022). However, the training regimen incorporated varied significantly across these studies [i.e., breath-holding protocol (static and/or dynamic), number of repetitions per session (5–10), number of sessions per week (4–7 days), and total training period (2–22 weeks)], precluding direct comparisons to be made. Yet it is noteworthy that all studies failing to record improvements in EPO and reticulocytes shared a common feature, that is the degree of desaturation attained by their subjects [85 ± 6%, Elia et al. (2019a); 84 ± 11%, Fernandez et al. (2019); 82 ± 7%, Bouten et al. (2022)], which was appreciably lower than in studies reporting favourable outcomes [73 ± 11%, de Bruijn et al. (2008); 73 ± 30%, Engan et al. (2013); 62 ± 10%, Elia et al. (2019a)]. Taken together, this retrospective analysis may suggest that the hypoxaemic-hypercapnic dose (i.e., exposure time and the degree of desaturation) most likely was inadequate to actuate erythropoiesis.

This supposition is partially supported by findings from intermittent hypoxic training studies (Gore et al. 2006; Wojan et al. 2021). For example, exposure to simulated altitudes of 4000–5500 m for 3 h per day, 5 days per week, led to a significant increase in EPO concentrations [(pre) 15.4 ± 8 mlU/L vs. (post) 31.3 ± 5.9 mlU/L], yet these elevations did not translate into measurable improvements in red cell volume or t-Hb_mass_ (Gore et al. 2006). These findings infer that a substantially greater hypoxaemic dose is necessary to effectively stimulate erythropoiesis, which may help explain the lack of observed changes in red blood cell count and t-Hb_mass_ in breath-hold training studies.

### Skeletal muscle capillarisation

Perhaps one of the least explored areas of breath-hold diving physiology is the skeletal muscle phenotypes of diving populations, with only a limited number of studies having delved into these using established wet-lab techniques (Bae et al. 2003; Park et al. 2005; Kjeld et al. 2018; Elia et al. 2019b). Interestingly competitive divers exhibited a higher capillary density, an increased capillary-to-fibre ratio, and a lower diffusion distance and sharing factor compared to matched non-diving cohorts (Elia et al. 2019b) (Fig. 1). These findings suggest that long-term breath-hold training may improve blood-to-tissue exchange capacity (Richardson et al. 1994; Saltin 1985). In part support of this conjecture is that hypoxia and muscle recruitment, particularly when combined, are evinced to serve a crucial role in the regulation and expression of vascular endothelial growth factor (VEGF) (Breen et al. 1996; Gustafsson and Sundberg 2000; Vogt et al. 2001), and consequently, the initiation of capillary neo-formation and angiogenesis (Arany et al. 2008; Desplanches et al. 1993; Kon et al. 2014; Terrados et al. 1990). It is conceivable therefore that the greater capillarisation noted in diving populations may be ascribed to their habitual activities, which involve frequent and repeated bouts of static and dynamic breath-holds.

*Ceteris paribus*, a greater capillary supply will improve the diffusive exchange of oxygen for oxidative phosphorylation, adenosine triphosphate re-synthesis, and the removal of metabolic waste products (e.g., carbon dioxide, ammonia, lactate) due to a greater capillary surface area in skeletal muscle and prolonged erythrocyte transit time (Krogh 1919; Andersen and Saltin 1985; Saltin 1985; Richardson et al. 1993, 1994, 1999). These combined effects can lead to improved muscular work rate, delay the onset of skeletal muscle fatigue, and ultimately extend time to task failure. Indeed, there is a large body of evidence demonstrating a strong link between capillarisation and exercise performance, with measures of capillary density positively correlating with training status, $\dot{\text{V}}$O_2max_, ventilatory threshold and critical power (Hermansen and Wachtlova 1971; Ingjer 1979; Robbins et al. 2009; Mitchell et al. 2018). Thence, if long-term breath-hold training effectively improves capillarisation, it could be appealing to athletic populations.

### Psychological resilience

A well-known aspect of breath-hold training is that it accentuates the magnitude of the diving response (see review by Elia et al. 2021c); however, a less-recognised benefit is its capacity to also improve psychological resilience (Rigg et al. 1974; Schagatay et al. 2000; Alkan and Akis 2013; Bourdas and Geladas 2024). Studies using cross-over designs with both trained and untrained subjects have shown that breath-hold divers are able to suppress their respiratory urges and extend their breath-holds far beyond their physiological breaking points (Bourdas and Geladas 2024). In contrast, untrained individuals tend to terminate their attempts at or near this point, suggesting a heightened capacity for stoicism among the trained divers. Furthermore, breath-hold divers exhibit greater resistance to stress, higher self-confidence, and lower negative affectivity (Alkan and Akis 2013). This ability to endure significant discomfort and persevere through mentally and physically challenging situations is essential for cultivating mental toughness—a trait strongly linked to success in sports [see review by Crust and Azadi (2010)]. This psychological construct is considered beneficial for competitive athletes (Crust and Clough 2005; Gucciardi et al. 2008; Connaughton et al. 2008), who frequently must push through both physical and psychological barriers during prolonged exertion (Crust and Azadi 2010). As such, breath-hold exercises could be propitious as a mental training strategy for developing and nurturing mental toughness in athletes.

### Exercise performance

It is well established that long-term breath-hold training leads to significant improvements in breath-hold performance (Elia et al. 2021a; Engan et al. 2013; Schagatay et al. 2000; Bourdas and Geladas 2021; Joulia et al. 2003), yet there is a paucity of research on its longitudinal effects on exercise performance (Bouten et al. 2022; Bourdas and Geladas 2021; Joulia et al. 2003). In particular, studies involving non-divers have shown that neither two or six weeks of daily dry static breath-holding (i.e., one set of five maximal attempts, each separated by a 2-min rest period), nor three months of simulated dynamic breath-hold training performed three times per week (i.e., repetition of 20 s breath-holds separated by 40 s of breathing room air during a 1-h steady state cycling exercise at 30% of $\dot{\text{V}}$O_2max_) were effective in improving exercise performance [i.e., $\dot{\text{V}}$O_2max_ and ventilatory threshold (Joulia et al. 2003); time to exhaustion test with intensity corresponding to 150% of PPO (Bourdas and Geladas 2021); 3-km cycling time-trial and $\dot{\text{V}}$O_2peak_ (Bouten et al. 2022)]. However, since none of these studies tracked breath-hold durations and/or desaturation levels across the training period, it remains unclear whether these results reflect the ineffectiveness of breath-holding as a hypoxic training modality, or if the hypoxaemic-hypercapnic dose was simply insufficient.

An additional method of breath-hold training that has gained interest in recent years is voluntary hypoventilation. Incorporating breath-holding into regular training sessions through voluntary hypoventilation has shown potential to improve athletic performance (Trincat et al. 2017; Karaula et al. 2016; Lavin et al. 2015; Woorons et al. 2016). This technique typically involves the athlete exhaling to a predetermined pulmonary volume (e.g., near functional residual capacity) and holding their breath for a set duration [e.g., 4–8 s; Woorons et al. (2020); Woorons et al. (2019); Woorons et al. (2008)], specific distance [e.g., 40-m sprint, Fornasier-Santos et al. (2018); 15-m, Trincat et al. (2017) or 25-m, Woorons et al. (2016) swim front crawl sprint] or until a strong urge to breathe is felt (Lavin et al. 2015) while continuing to exercise. Thenceforth, the athlete exhales the remaining air and undergoes a brief recovery period whilst breathing ad libitum [30 s, Fornasier-Santos et al. (2018); 16 s, Woorons et al. (2020); 24 s, Lapointe et al. (2020)], before repeating the exhale-hold cycle. This combination of repeated-sprint training with short bouts of end-expiratory breath-holding, similarly to dynamic breath-holds (Joulia et al. 2003; Elia et al. 2021b), is effective in inducing hypoxaemic (~ < 88%) and hypercapnic stress (Yamamoto et al. 1987, 1988; Dempsey and Wagner 1999; Findley et al. 1983). Over time, this training method appears effective in improving swimming performance [twice per week for 5-weeks; Woorons et al. (2016)], running economy [three times per week for 4-weeks; Lavin et al. (2015)] and repeated-sprint ability [six sessions performed over a 2-week period; Trincat et al. (2017)]. While the precise mechanism(s) driving these improvements remain(s) unclear, the authors have attributed them to an enhanced buffering capacity and pH regulation, and to a better oxygen utilisation in fast-twitch muscle fibres (Bishop et al. 2011).

## Safety and adverse effects

It is imperative that practitioners are well-versed with the inherent risks of this training practice. Notably, prolonged breath-holding may lead to a hypoxic blackout (i.e., loss of consciousness) and, depending on whether this occurs under land- or water-based activities, it can lead to injury (e.g., fall-related), drowning and even be fatal. It is, thus, paramount that stringent safety measures are implemented when breath-hold-related activities are practiced. Specifically, breath-holding must never be carried out without direct supervision and, where possible, pulse oximeters^1^ should be used to monitor the oxygen levels of the apnoeist. Also, it is crucial that a safety threshold is established, and if this level is reached, supervisors must promptly instruct the apnoeist to abort their attempt and resume normal breathing. Incorporating these rather simple but essential measures will aid towards mitigating any adverse events.

A comprehensive understanding of factors that could also potentiate the risk of suffering from a hypoxic blackout is also key for ensuring the safety and well-being of apnoeists. In this regard, evidence suggest that breath-holding activities incorporating an exercise component [i.e., dynamic breath-holds; Lindholm (2007); Elia et al. (2021b)], pre-breath-hold hyperventilation (Elia et al. 2024b; Craig 1976, 1961), exercise (Lindholm and Gennser 2005) and fasting (Elia et al. 2022a, 2024b) have all been shown to exacerbate this risk; especially over a series of repeated attempts. Therefore, it is important that these risk factors are not overlooked but rather be carefully considered when designing, prescribing, and/or engaging in breath-hold-related activities.

### Health implications

Information pertaining to the possible health implications associated with long-term engagement in breath-hold-related activities is currently limited [see review by Elia et al. (2021c)]. Preliminary evidence suggests that while sustained breath-holding may pose ramifications for renal health (Oh et al. 2017), it appears to have no adverse effects on cardiac health or vascular integrity (Zelenkova and Chomahidze 2016; Tanaka et al. 2016; Doerner et al. 2018). Conversely, the long-term effects on bone tissue (Hwang et al. 2006; Kjeld et al. 2018; Seo et al. 2018), the central nervous system (Doerner et al. 2018; Kohshi et al. 2014; Potkin and Uzsler 2006), and neurocognition function (Ridgway and McFarland 2006; Ratmanova et al. 2016; Steinberg and Doppelmayr 2019; Billaut et al. 2018; Allinger et al. 2024a; Elia et al. 2022b, 2024a) remain less clear. This limited body of research highlights the need for further cross-sectional and longitudinal studies aimed at deepening our understanding of the potential maladaptation(s) associated with long-term breath-hold training.

## Conclusion

This review provided (*i*) an update of the physiological responses associated with acute and long-term engagement in breath-hold-related activities relevant to athletic performance, (*ii*) delved into breath-hold priming strategies and training regimens used to improve performance, (*iii*) offered guidance on key factors to consider when designing and administering breath-hold-related activities, and (*iv*) outlined how breath-holding can safely and effectively be applied in athletic pursuits. The current evidence suggests that whilst the potential application of breath-holding may be propitious, further placebo-controlled studies are needed to thoroughly assess its efficacy as a priming strategy. In addition, it is evident that developing an effective protocol (i.e., type of breath-holding, load, repetitions, duration etc.) and administering it successfully is more complex than initially thought, with several factors requiring careful consideration and highlighting the need for adaptable, and context-specific approaches to integrating breath-holding into athletic preparation. Finally, while dynamic breath-hold training shows the greatest potential as a performance optimisation strategy, additional research is needed to establish the optimal training protocol, including the appropriate hypoxaemic-hypercapnic dose and duration.

## Abbreviations

EPO: Erythropoietin
HIF: Hypoxia-inducible factor
IBM: Involuntary breathing movements
PPO: Peak power output
SpO_2_: Peripheral oxygen saturation
$$\dot{\text{Q}}$$: Cardiac output
t-Hb_mass_: Total haemoglobin mass
TLC: Total lung capacity
VEGF: Vascular endothelial growth factor
$$\dot{\text{V}}$$O_2_: Oxygen uptake
$\dot{\text{V}}$O_2max_: Maximal oxygen uptake
$\dot{\text{V}}$O_2peak_: Peak oxygen uptake

## Footnotes

1 Low-cost pulse oximeters have become increasingly available in the market; however, their accuracy is often not validated. Therefore, it is recommended to use units cleared by the International Organisation for Standardisation, which have been tested and certified to be accurate to root mean square error of < 3% at oxygen saturation levels between 70 and 100% (see review by Shi et al. 2022).
